# Vulnerability Reduction Needed to Maintain Current Burdens of Heat-Related Mortality in a Changing Climate—Magnitude and Determinants

**DOI:** 10.3390/ijerph14070741

**Published:** 2017-07-07

**Authors:** Christofer Åström, Daniel Oudin Åström, Camilla Andersson, Kristie L. Ebi, Bertil Forsberg

**Affiliations:** 1 Division of Occupational and Environmental Medicine, Department of Public Health and Clinical Medicine, Umeå University, SE90187 Umeå, Sweden; daniel.oudin.astrom@umu.se (D.O.A.); bertil.forsberg@envmed.umu.se (B.F.); 2 Department of Clinical Sciences, Malmö, Lund University, Jan Waldenströms gata 35, SE21428 Malmö, Sweden; 3 Swedish Meteorological and Hydrological Institute, Folkborgsvägen 17, SE60176 Norrköping, Sweden; camilla.andersson@smhi.se; 4 School of Public Health, University of Washington, 4225 Roosevelt Way NE #100, Seattle, WA 98105, USA; krisebi@uw.edu

**Keywords:** adaptation, climate change, health, heat, Europe

## Abstract

The health burden from heatwaves is expected to increase with rising global mean temperatures and more extreme heat events over the coming decades. Health-related effects from extreme heat are more common in elderly populations. The population of Europe is rapidly aging, which will increase the health effects of future temperatures. In this study, we estimate the magnitude of adaptation needed to lower vulnerability to heat in order to prevent an increase in heat-related deaths in the 2050s; this is the Adaptive Risk Reduction (ARR) needed. Temperature projections under Representative Concentration Pathway (RCP) 4.5 and RCP 8.5 from 18 climate models were coupled with gridded population data and exposure-response relationships from a European multi-city study on heat-related mortality. In the 2050s, the ARR for the general population is 53.5%, based on temperature projections under RCP 4.5. For the population above 65 years in Southern Europe, the ARR is projected to be 45.9% in a future with an unchanged climate and 74.7% with climate change under RCP 4.5. The ARRs were higher under RCP 8.5. Whichever emission scenario is followed or population projection assumed, Europe will need to adapt to a great degree to maintain heat-related mortality at present levels, which are themselves unacceptably high, posing an even greater challenge.

## 1. Introduction

Ambient temperature, particularly extremes thereof, affects people’s health in most parts of the world. Individuals and communities in many European countries have experienced adverse health impacts from exposure to high ambient temperatures over the last 15 years [1,2,3]. Although studies found that population vulnerability to extreme heat has decreased over recent decades as global mean surface temperatures increased [4,5,6], other studies found unclear patterns [1]. Whether vulnerability will decline over future decades is an open question. Reasons typically mooted for any decline in vulnerability are increased access to air conditioning, individual acclimatization, changes to the built environment, and perhaps early warning and response systems. Vulnerability trends, and their interactions with climate change, will be a key driver of the magnitude and pattern of adaptation needed to increase local and national resilience to climate change.

The frequency, intensity, and duration of temperature extremes are projected to increase with climate change [7,8], with associated increases in morbidity and mortality. The warming of the climate in the coming century will be faster than in the last, which means that future populations might not be able adapt at the same rate as past populations [6].

### Hypothesis and Aim

As climate change progresses, the extent to which adaptation will be needed to protect and promote population health will likely increase. Different measures will be plausible and preferable in different countries because of underlying factors such as demographics, prevalence of chronic disease, appropriateness of societal infrastructure for higher temperatures, governance structures, and other factors. Within this context, we estimate for European regions how much adaptation would be needed, in different age-groups, to lower vulnerability under different scenarios of climate change in order not to see an increase in heat-related deaths.

## 2. Materials and Methods

### 2.1. Climate Scenarios

Temperature and humidity were extracted from 18 future climate realizations from the Coordinated Regional Climate Downscaling Experiment (EURO-CORDEX) scenarios for Europe [9]. For these climate scenarios, dynamical downscaling was conducted with the Rossby Centre regional atmospheric model RCA4 [10], forced on the boundary by nine global climate models (GCMs) and two future greenhouse gas emission scenarios. Greenhouse gas emissions evolve based on the representative concentration pathways (RCPs) [11,12,13]: RCP 4.5 [11,14] and RCP 8.5 [12,15] from 2006 and onward, and by historical conditions for the period before that. Both were assessed by the Intergovernmental Panel on Climate Change (IPCC) [16], where RCP 8.5 is a pessimistic scenario and RCP 4.5 is more optimistic. The climate scenarios used, based on these emission scenarios, are described in detail in [10] and [17]. The period 1981–2010 was chosen as the reference period and the period 2036–2065 was chosen to project future risks. The climate data have a resolution of approximately 50 × 50 km covering Europe.

An evaluation of RCA4 [10] forced by observed meteorology [18] in the present climate shows small differences in temperature compared with observations, but with a slightly over-enhanced seasonal temperature cycle. The ensemble mean of the RCA4 downscaling of the nine GCMs is only 0.5 °C colder than RCA4 forced by measurements, but with large variation between the ensemble members (from −2 °C to 0.5 °C compared to RCA4 forced by measurements).

### 2.2. Population Data

The population data and future scenarios were gridded datasets at 50 × 50 km compiled in the Integrated Assessment of Health Risks of Environmental Stressors in Europe (INTARESE) and Health and Environment Integrated Methodology and Toolbox for Scenario Development (HEIMSTA) projects [19]. The data were divided into 5-year age groups and separated by gender up to 65 years of age where data were aggregated into a 65+ age group. A scenario assuming a medium population growth rate was used for 2050. To estimate exposure to high ambient temperature, the climate grid cell closest to each population grid cell was assigned as the exposure for that population. The population data were limited to 29 countries in the European region.

### 2.3. Impact Calculation

We estimated to what degree overall adaptation, biological or societal, would be needed to reduce the health impacts of heat to maintain heat-related mortality at a present-day level. The extent of adaptation needed was calculated from increased exposure and changing population. To estimate impacts, we applied the exposure-response relationships from a European multi-city study [1]. In that study, the estimated relative risk was calculated by comparing heat-related mortality at the 75th and 99th percentile of summer mean temperatures.

For each of the population grids, we calculated the 75th and the 99th percentiles of the daily mean summer temperatures for each of the nine climate models for the baseline period 1981–2010. For day *i* in grid *j* an exposure *E_ij_* was calculated by assigning 0 to all temperatures below the 75th percentile, and 1 if the temperature equaled the 99th percentile. Because the epidemiological study only compared the change in mortality between the two temperature thresholds, it was not clear how to estimate the risk increases between the temperature intervals. By assuming a linear increase in relative risk between the percentiles and beyond, temperatures above the 75th percentile were assigned values accordingly. The impact in grid *j* was then calculated as:

(1) Impactj =∑i=1nEij* PopjPopR

where *E_ij_* is the exposure measure in grid *j* at time *i, Pop_j_* is the population in grid *j* and *Pop_R_* is the population in the region to which the estimates were weighted by and aggregated to. This results in a grid of calculated impacts for Europe. The estimates were calculated for each of the 29 countries in the study, as well as for Northern, Western, Southern and Eastern regions of Europe per the United Nation definition of the European regions from the Standard Country or Area Codes for Statistics Use [20].

As a baseline, the calculated mortality impacts from present-day climate were used. The future projections were based on changes in climate, population and demographic composition, jointly and separately to estimate the individual effects of each. These impacts were then compared with the baseline estimates to calculate the extent of adaptation needed to maintain the current level of heat-related mortality with higher temperatures and different populations. The analyses were carried out considering the change in total population, as well as changes in the population above 65 years of age only. This was done to estimate the adaptation needs among the elderly population, who are more vulnerable to high ambient temperatures.

We calculated an Adaptive Risk Reduction (ARR) based on the differences between present and future impacts, calculating the percent reduction of the future impacts required to have the same burden of heat-related mortality as the present period.

(2) ARR = 1 − ImpactPImpactF

## 3. Results

Southern Europe will have the largest increase in heat-related morality in the 2050s due to higher temperatures, with smaller increases in the rest of Europe. Total population numbers and demographic change affected regional differences. Country-level projections are shown in Table S1 in the supplementary material.

On average, the population projections for the future yield a very minor negative ARR (less deaths than without additional adaption) of −0.2% for Europe as a whole in a scenario where no further climate change occurs (Table 1). These results were mainly driven by the projected population decrease in Eastern Europe, although other regions were projected to have an increased need for adaptation. For Eastern Europe, the impact was projected to decrease and the ARR was estimated to be −17.2% due to population change alone.

The projected ARR with climate change and demographic change suggests that additional adaptation will be needed in all parts of Europe to maintain current levels of heat-related mortality. RCP 8.5 shows a slightly larger range, with a small shift towards a higher estimated ARR. The differences between regions within each RCP was much larger than between RCPs.

The estimated ARR for the entire population of Europe was 47.0% (33.7% to 54.9%) and 53.4% (34.6% to 63.7%) for RCP 4.5 and RCP 8.5, respectively; the ranges only reflect the uncertainty in the climate change projections that were used (Table 1). This means that adaptation needs to be ramped up in Europe to lower heat-related vulnerability to present day levels. If this does not occur, then heat-related mortality would be expected to increase. For Europe on average, population change will have a minor effect on the results with an estimated ARR of 47.0% without population change and 46.8% with population change.

The results differ across regions (Table 1). The greatest need for reduction in vulnerability will be in Southern Europe, with estimated ARRs of 53.5% and 60.8% for RCP 4.5 and RCP 8.5 respectively. Adding population change, we estimated the ARRs to be 54.8% and 61.7%. For Eastern Europe, the region with the least need for adaptation, the estimated ARRs were 45.6% and 49.8% compared with 36.6% and 41.7% when population change was accounted for. The results also vary across the climate models. Under RCP 4.5, the estimates for Eastern Europe ranged from 18.0 to 44.1%, depending on what climate model was used.

Demographic change in Europe, with the greatest changes in the older age groups (Table 2), will increase the need for adaptation regardless of climate change. The estimated ARR for Europe was 38.9% for the part of the population above 65 years of age. Demographic change also will affect the estimated ARR differently in different regions. For Eastern Europe, the demographic shift to a more elderly population is quite marked and the ARR for total population, not taking climate change into account, was estimated to be negative. The estimated ARR for the group 65 years of age and older was 38%.

Overall, the estimated ARR for the elderly in a changed climate ranged from 64.1 to 74.7% for RCP 4.5 and 69.3 to 78.5% for RCP 8.5, in the different regions. The estimated ARR of 78.5% for the elderly population in Southern Europe means that future heat-related mortality must be reduced by adaptation to a fifth to be kept at current levels.

## 4. Discussion

Future climate is expected to considerably increase the need for adaptation in European countries until at least the 2050s. The range of estimates between models within each RCP were greater than between the RCPs. This was expected, because the future time period analyzed is within the climate change commitment period, such that the differences between RCPs will be more pronounced towards the end of the century. The estimated ARR for the different projections suggest that, in general, European countries need to lower population vulnerability to heat to about a half of current levels just to keep heat-related mortality at present day levels. This also means that if a reduction in mortality is to be accomplished, then an even larger reduction is needed. As this is the aim for many countries, large adaptation efforts are needed.

We chose not to calculate the number of deaths attributable to heat, or to calculate the increase in mortality due to heat in the 2050s; there are many such projections [21,22,23,24]. The estimated ARR is an approach to estimate the magnitude of challenges climate change could pose to future populations and sub-groups. Trends in population characteristics that could exacerbate or ameliorate the heat effect, as well as the possibilities and limitations of heat-related interventions, will vary between countries, regions, and cities. Understanding the possible magnitude of future adaptation needs could facilitate identifying interventions to increase population health resilience in a future climate.

Investigating the effects of climate change and demographics separately, climate appears to be the main driver of the future heat burden. Demographic change will also increase the need for adaptation by a similar order of magnitude.

Projections for the future population of Europe indicate a demographic composition by mid-century where 28.1% of the population will be above 65 years of age. In January 2015, the proportion was 18.9%. This was reflected by a considerably higher projected ARR for this age group in the 2050s. This indicates the need for age-specific analyses when estimating future health risks to provide more accurate projections of the risks of increasing temperatures. A next step would be to account for factors such as the prevalence of chronic diseases and mortality patterns that alter vulnerability to heat. However, limited data at local, regional, and even national scales make this challenging to model. On the other hand, heat-related mortality rates in older adults are a proxy for some chronic diseases that increase vulnerability to heat. A study of the future prevalence of diabetes for 21 European countries of the population aged 20–79 [25] estimated an average increase in diabetes prevalence of 13%, and an increase in the number of individuals by 23% by 2035 compared to 2013. This will likely increase the health burden from heat because people diagnosed with diabetes are more vulnerable [26]. The increase in the number of patients diagnosed with a certain disease should be treated with caution, as an increase in number (prevalence) might be due to higher survival rates [27]. Persons diagnosed with Chronic Obstructive Pulmonary Disease (COPD) are also among the most vulnerable to heat, and the increase in prevalence is expected to continue [28]. In some countries, however, the upward trend of COPD prevalence may have reached a plateau as a result of smoking prevention [29]. How future societies manage the burden of chronic diseases will have a considerable impact on population vulnerability to heat.

The combined effects of demographic and climatic changes highlight the considerable increased needs for adaptation in Europe. In Southern Europe under RCP 8.5, vulnerability would need to be decreased to a fifth for the age group above 65 years if heat-related mortality rates are to remain at today’s level.

**Figure 1:**
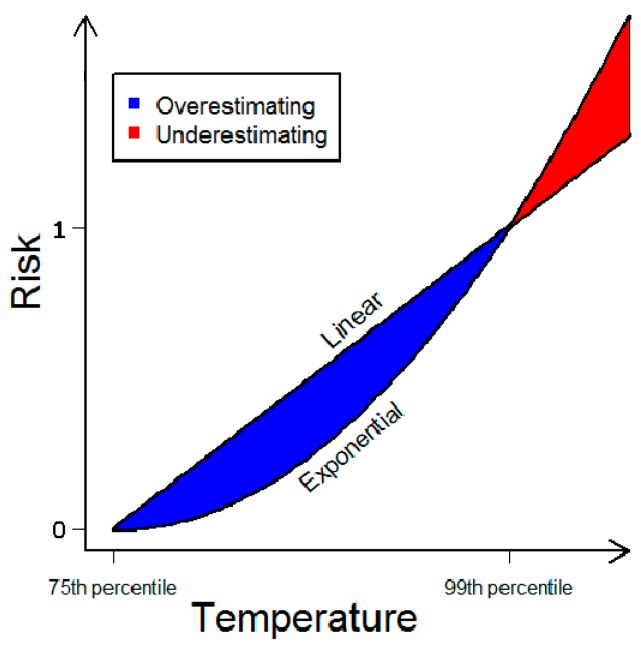
The difference between using a linear and an exponential relationship to describe heat-related mortality, assuming that the exponential relationship is the correct one.

The relative risks from the multi-city study were calculated as the difference in risk at the 75th and the 99th percentile of the mean daily summer temperature. The assumption that the risk increase is linear between the two introduces uncertainty. Temperature-mortality relationships generally have a temperature at which mortality is at its minimum. If this temperature is lower than the 75th percentile, then this method would classify fewer days with increased mortality. If the optimal temperature is higher than the 75th percentile, then more days would be classified as days with increased heat-related mortality. We assumed that any misclassifications of this kind would be equally present in the baseline and future period, and the possible errors would cancel each other out. In addition, the assumed linear increase could also increase uncertainty in the estimates. Most recent studies find that temperature-mortality relationships follow an exponential trend [30]. Applying a linear estimate instead of an exponential could overestimate the risk between the 75th and 99th percentile and underestimate the impacts above the 99th percentile (Figure 1). As temperatures shift to warmer and more extreme days, both intervals are expected to increase, with the relative increase likely to be larger for the warmest interval. Assuming a linear temperature-mortality relationship would potentially underestimate the true impacts of climate change on heat-related mortality [31].

The future burden is driven partially by future demographics. Different population projections will estimate similar numbers in the 65+ age group because everyone expected to be older than 65 in the 2050s is alive now. Immigration could alter this in some countries, but it is unlikely that people migrating from now until 2050 would alter the number of people 65+ by very much. The sheer number of elderly in the future will increase the size of the vulnerable population; this is reflected in the projection that the ARR would be around 40% even without climate change.

The need for additional adaptation is clear from our results. To what degree people, populations and societies will be able to adapt is uncertain. The fraction of mortality attributable to heat is larger in the two more southern countries in the study, Spain and Italy, than in Sweden and the United Kingdom [30]. These results suggest that living in warmer conditions do not necessarily increase heat resilience. Another study of European conditions [1] showed that estimated risks for the Southern and Mediterranean cities were generally higher than for Northern and Western Europe. These results indicate that how future populations will react to a higher temperature is hard to project.

Studies reach conflicting conclusions on how socioeconomic factors could act as risk-modifiers for heat-related health issues. Studies from the United States (US) found that a high prevalence of air conditioning in a population lowers heat vulnerability. The epidemiological evidence is ambiguous on how socioeconomic factors alter the impact of high temperatures. A systematic review looking at vulnerability factors for heat-related mortality [32] found inconsistent results in the European studies. High socioeconomic status (SES) on an ecological level was found to have a protective effect in Paris compared to low SES [33], while the opposite was found in London [34]. Two studies investigating the effect of SES in England, Wales, and Stockholm found no difference between high and low SES [35,36]. In Italy, high SES had a stronger protective effect than in other parts of Europe [37]. This lack of consensus makes it hard to incorporate key SES variables in an analysis on a European scale. Further, these types of data are usually collected at national or regional scales and are not necessarily comparable between countries.

The shared socio-economic pathways (SSPs) describe changes in factors that might alter the exposure and vulnerability of future populations to high temperatures; further exploration of ARRs under the SSPs is warranted. As a step in that direction, some of the main drivers of the health burdens associated with high temperatures are shown in Table 3 along with how the SSPs might change the impact of these drivers. No SSP is universally better than the others. The differences across the SSPs mean that they should be considered when projecting future health impacts.

Large adaptation needs are identified in this study. There is, however, limited evidence to support selecting interventions to lower heat-related health issues. The World Health Organization (WHO) issued recommendations for individual short-term adaptation [38]. These include advice on maintaining the living space cool, keeping out of the heat and keeping your body cool and hydrated. One adaptation measure suggested is the use of air conditioning to lower exposure to high temperatures. Increases in home air conditioning prevalence has lowered heat-related mortality in the US [39]. However, there are limited studies from Europe on the effectiveness of air conditioning, because its use is sparse in most parts of Europe. Such an intervention is also energy-consuming and can increase the urban heat island. There is a clear upper limit to this type of intervention. Heatwave early warning systems have been shown to have a protective effect in a number of studies [40,41,42,43,44], and such systems are one of the more obvious adaptation measures to lower short-term effects.

Apart from changing people’s behavior to lower their exposure to heat, changes to the built environment can also lower people’s exposure. Increasing the albedo of buildings, shading and passive cooling are interventions that are potentially energy-efficient and possible to implement in existing buildings. Doing so would lower indoor temperatures and reduce urban heat island effects. In the long-term, urban planning, building, and land-use regulations could be the most effective way to lower temperature exposure. The long lead time of such interventions will, however, require political will. As urbanization trends continue, with more people living in urban areas, lowering the temperature in such environments would decrease population vulnerability to some extent.

## 5. Conclusions

Countries in Europe are striving to lower the current health burden from heat by introducing heatwave early warnings systems, and revising health care plans to take ambient temperatures into account. Whichever climate scenario is followed, whichever population projection assumed, Europe will need to adapt to a greater degree to maintain heat-related mortality at present-day levels, which are themselves unacceptably high, posing an even greater challenge.

## Figures and Tables

**Table 1:** Estimated Adaptive Risk Reduction (ARR) (percent) needed for four European regions to maintain current levels of risk, comparing present and future populations (all ages and adults over 65 years of age), and present and projected climate in the 2050s under Representative Concentration Pathway (RCP) 4.5 and RCP 8.5.

		All Ages		Above Age 65
	Region	Present Population	Future Population	Future Population
Present climate	All study area		−0.2 (−0.2, −0.2)	38.9 (38.9, 38.9)
	Northern Europe		2.2 (2.1, 2.2)	34.7 (34.7, 34.7)
	Western Europe		7.0 (7.0, 7.0)	41.3 (41.3, 41.3)
	Eastern Europe		−17.2 (−17.2, −17.2)	38.0 (38.0, 38.0)
	Southern Europe		3.4 (3.4, 3.4)	45.9 (45.9, 45.9)
RCP 4.5	All study area	47.0 (33.7, 54.9)	46.8 (33.3, 54.4)	67.5 (59.4, 72.3)
	Northern Europe	45.0 (31.7, 54.6)	46.1 (32.4, 55.2)	64.1 (54.9, 70.1)
	Western Europe	45.1 (29.7, 53.3)	48.9 (34.5, 56.4)	67.8 (58.7, 72.5)
	Eastern Europe	45.6 (29.5, 52.1)	36.6 (18, 44.1)	66.6 (56.8, 70.6)
	Southern Europe	53.5 (44.4, 61.2)	54.8 (46, 62.3)	74.7 (69.7, 78.9)
RCP 8.5	All study area	53.4 (34.6, 63.7)	52.9 (34, 63.2)	71.4 (59.8, 77.7)
	Northern Europe	53.6 (44, 62.9)	54.3 (44.9, 62.9)	69.5 (63.2, 75.1)
	Western Europe	50.1 (24.6, 61.4)	53.4 (29.8, 64)	70.7 (55.8, 77.3)
	Eastern Europe	49.8 (32.1, 58.4)	41.7 (21.6, 51.7)	69.3 (58.6, 74.6)
	Southern Europe	60.8 (46.7, 68.7)	61.7 (47.9, 69.5)	78.5 (70.7, 82.9)

The results are the mean for nine climate models. The range of the estimates built on the different climate models is presented in brackets.

**Table 2:** Changes in population in Europe and the four sub-regions (%).

	Population change	
Region	All Ages	Above 65
All study area	−0.2%	63.3%
Northern Europe	13.8%	61.9%
Western Europe	−1.7%	54.0%
Eastern Europe	−14.8%	68.4%
Southern Europe	1.8%	74.8%

**Table 3:** Different drivers of heat-related mortality and how they could change under three socio-economic pathways (SSPs). Cells marked with green indicate a lowering of the health impacts and red an increase.

Drivers	SSP2	SSP1	SSP3
Population	Medium	Low	Low
Age-structure		Larger proportion of elderly	Smaller proportion of elderly
Chronic disease prevalence		Higher, better care	Higher, poor care
Urbanization	Medium	High	Low
Access to indoor cooling	Medium	High	Low
Urban planning	Continuation of historical patterns	Well managed	Poorly managed
Heatwave Early Warning System	Medium	High	Low
Societal participation	Medium	High	Low
Equity	Medium	High	Medium

## Supplementary Materials

The following are available online at www.mdpi.com/1660-4601/14/7/741/s1, Table S1: Estimated Adaptive Risk Reduction (ARR) (%) needed for four European countries to maintain current levels of risk, comparing present and future populations (all ages and adults over 65 years of age), and present and projected climate in the 2050s under Representative Concentration Pathway (RCP) 4.5 and RCP 8.5.
