# Supplementary material for: Vulnerability Reduction Needed to Maintain Current Burdens of Heat-Related Mortality in a Changing Climate—Magnitude and Determinants
Source: Int J Environ Res Public Health. 2017 Jul 7;14(7):741. doi: 10.3390/ijerph14070741 (PMC5551179; doi:10.3390/ijerph14070741)
Supplement: Supplementary file 1 [file ijerph-14-00741-s001.pdf]

Country	Present climate Future population			RCP4.5 Present population			RCP4.5 Future population		
	Mean	Max	Min	Mean	Max	Min	Mean	Max	Min
Austria	1,5%	1,5%	1,5%	49,3%	59,6%	32,5%	50,0%	60,2%	33,5%
Belgium	6,9%	6,9%	6,9%	44,8%	56,1%	30,7%	48,6%	59,1%	35,5%
Bulgaria	-39,0%	-39,0%	-39,0%	54,8%	66,4%	42,4%	37,2%	53,3%	19,9%
Switzerland	10,8%	10,8%	10,8%	52,4%	61,7%	37,9%	57,6%	65,8%	44,6%
Cyprus	25,3%	25,3%	25,3%	61,2%	70,0%	53,5%	71,0%	77,5%	65,3%
Czech Republic	-1,3%	-1,3%	-1,3%	46,7%	57,5%	28,4%	46,0%	57,0%	27,4%
Germany	-15,8%	-15,8%	-15,8%	46,7%	56,6%	32,0%	38,2%	49,7%	21,3%
Denmark	1,2%	1,2%	1,2%	49,0%	61,0%	37,2%	49,6%	61,5%	38,0%
Estonia	-8,5%	-8,5%	-8,6%	48,4%	58,1%	31,8%	44,0%	54,5%	25,9%
Spain	11,7%	11,7%	11,6%	56,7%	66,2%	46,4%	61,7%	70,1%	52,6%
Finland	1,9%	1,9%	1,8%	48,6%	60,0%	36,5%	49,5%	60,7%	37,7%
France	7,9%	8,0%	7,8%	50,0%	58,1%	37,8%	54,0%	61,4%	42,8%
Greece	-2,1%	-2,0%	-2,1%	58,3%	68,9%	49,8%	57,5%	68,2%	48,7%
Hungary	-11,7%	-11,7%	-11,7%	47,3%	57,8%	30,5%	41,1%	52,8%	22,3%
Ireland	27,0%	27,1%	27,0%	46,0%	63,2%	29,6%	60,6%	73,2%	48,6%
Italy	-5,2%	-5,1%	-5,2%	56,3%	65,9%	44,5%	54,0%	64,1%	41,6%
Lithuania	-26,4%	-26,4%	-26,4%	44,8%	55,1%	21,2%	30,2%	43,2%	0,4%
Luxembourg	33,2%	33,2%	33,2%	47,5%	56,2%	32,6%	64,9%	70,7%	54,9%
Latvia	-20,7%	-20,7%	-20,7%	45,3%	56,4%	22,5%	34,0%	47,4%	6,5%
Malta	3,5%	3,5%	3,5%	64,8%	73,6%	55,5%	66,0%	74,5%	57,0%
Netherlands	4,3%	4,3%	4,3%	45,8%	57,1%	35,0%	48,2%	58,9%	37,8%
Norway	18,3%	18,3%	18,3%	48,4%	64,3%	33,8%	57,8%	70,8%	45,9%
Poland	-18,7%	-18,7%	-18,7%	44,8%	54,0%	24,3%	34,5%	45,4%	10,1%
Portugal	-6,6%	-6,4%	-6,7%	48,4%	56,1%	37,6%	45,1%	53,4%	33,7%
Romania	-22,6%	-22,6%	-22,6%	51,9%	63,1%	36,0%	41,1%	54,7%	21,5%
Sweden	12,1%	12,1%	12,1%	50,5%	61,0%	39,3%	56,5%	65,7%	46,7%
Slovenia	-2,8%	-2,8%	-2,8%	51,7%	62,9%	33,7%	50,3%	61,8%	31,8%
Slovakia	-10,0%	-9,9%	-10,0%	47,8%	57,5%	29,8%	42,5%	53,3%	22,7%
United Kingdom	14,4%	14,5%	14,4%	46,2%	59,5%	30,5%	54,0%	65,4%	40,5%
Mean	-0,4%	-0,4%	-0,4%	50,2%	60,8%	35,6%	49,9%	60,5%	35,0%

	RCP8.5 Present population			RCP4.5 Future population		
Country	Mean	Max	Min	Mean	Max	Min
Austria	51,8%	65,6%	27,5%	52,6%	66,1%	28,5%
Belgium	51,1%	63,5%	28,0%	54,4%	66,0%	33,0%
Bulgaria	61,9%	70,4%	52,1%	4,7%	58,8%	33,4%
Switzerland	56,4%	68,4%	32,2%	61,1%	71,8%	39,5%
Cyprus	69,9%	76,6%	60,2%	77,5%	82,5%	70,2%
Czech Republic	48,7%	64,6%	25,0%	48,0%	6,4%	2,4%
Germany	49,7%	65,2%	25,6%	41,8%	59,7%	13,9%
Denmark	55,4%	68,8%	40,7%	56,0%	69,2%	41,4%
Estonia	56,1%	65,5%	41,6%	5,2%	62,6%	3,7%
Spain	63,8%	73,3%	49,5%	68,0%	76,4%	55,3%
Finland	56,5%	65,8%	40,1%	57,3%	66,5%	41,2%
France	56,3%	67,0%	36,0%	59,8%	69,6%	41,1%
Greece	65,8%	73,8%	57,4%	65,1%	7,3%	56,4%
Hungary	49,0%	62,1%	24,2%	43,1%	57,7%	15,3%
Ireland	58,7%	71,4%	43,7%	69,8%	79,1%	58,9%
Italy	61,4%	71,7%	42,0%	59,4%	70,2%	3,9%
Lithuania	51,4%	64,2%	37,2%	38,6%	54,8%	20,6%
Luxembourg	52,7%	65,8%	25,8%	68,4%	77,1%	50,4%
Latvia	52,6%	63,7%	39,8%	42,8%	56,3%	27,4%
Malta	71,9%	79,3%	60,2%	72,9%	8,0%	6,2%
Netherlands	52,7%	65,6%	33,7%	54,7%	67,1%	36,5%
Norway	57,1%	68,7%	39,9%	65,0%	74,4%	5,1%
Poland	47,6%	61,7%	27,8%	3,8%	54,6%	14,3%
Portugal	57,2%	66,1%	50,4%	54,4%	63,9%	47,1%
Romania	57,8%	67,0%	45,3%	48,3%	5,9%	33,0%
Sweden	57,6%	68,1%	43,3%	62,7%	71,9%	50,2%
Slovenia	54,1%	67,7%	27,1%	52,8%	66,8%	25,0%
Slovakia	50,2%	63,0%	28,9%	45,3%	59,3%	2,2%
United Kingdom	57,2%	67,8%	44,5%	63,4%	72,5%	52,5%
Mean	56,3%	67,7%	39,0%	51,6%	58,7%	31,3%
